# Adaptive image encryption approach using an enhanced swarm intelligence algorithm

**DOI:** 10.1038/s41598-025-86569-9

**Published:** 2025-03-19

**Authors:** Sachin Minocha, Suvita Rani Sharma, Birmohan Singh, Amir H. Gandomi

**Affiliations:** 1 School of Computer Science Engineering and Technology, Bennett University, Greater Noida, India; 2 Department of Computer Science and Engineering, Sant Longowal Institute of Engineering and Technology, Longowal, India; 3 Faculty of Engineering & IT, University of Technology Sydney, Sydney, Australia; 4 University Research and Innovation Center (EKIK), Obuda University, Budapest, Hungary; 5 Department of Computer Science, Khazar University, 41 Mahsati, Baku, Azerbaijan

**Keywords:** Hippopotamus optimization, Iterative Cosine Operator, Chaotic, PWCM, PWLCM, Image encryption, Medical image, Hyperspectral image, Computational science, Computer science

## Abstract

Chaos-based encryption methods have gained popularity due to the unique properties of chaos. The performance of chaos-based encryption methods is highly impacted by the values of initial and control parameters. Therefore, this work proposes Iterative Cosine operator-based Hippopotamus Optimization (ICO-HO) to select optimal parameters for chaotic maps, which is further used to design an adaptive image encryption approach. ICO-HO algorithm improves the Hippopotamus Optimization (HO) by integrating a new phase (Phase 4) to update the position of the hippopotamus. ICO-HO updates the position of hippopotamuses using ICO and opposition-based learning, which enhances the exploration and exploitation capabilities of the HO algorithm. ICO-HO algorithm’s better performance is signified by the Friedman mean rank test applied to mean values obtained on the CEC-2017 benchmark functions. The ICO-HO algorithm is utilized to optimize the parameters of PWLCM and PWCM chaotic maps to generate a secret key in the confusion and diffusion phases of image encryption. The performance of the proposed encryption approach is evaluated on grayscale, RGB, and hyperspectral medical images of different modalities, bit depth, and sizes. Different analyses, such as visual analysis, statistical attack analysis, differential attack analysis, and quantitative analysis, have been utilized to assess the effectiveness of the proposed encryption approach. The higher NPCR and UACI values, i.e., 99.60% and 33.40%, respectively, ensure security against differential attacks. Furthermore, the proposed encryption approach is compared with five state-of-the-art encryption techniques available in the literature and six similar metaheuristic techniques using NPCR, UACI, entropy, and correlation coefficient. The proposed methods exhibit 7.9995 and 15.8124 entropy values on 8-bit and 16-bit images, respectively, which is better than all other stated methods, resulting in improved image encryption with high randomness.

## Introduction

Recent growth in digital information technology has led to the transmission of many sensitive and confidential images over public networks. The availability of such crucial information over public networks raises concerns for image security. Image security can be achieved using steganography, watermarking, and encryption. Image steganography hides the secret data under another image, video, or audio. In this technique, only the intended recipient and the sender are aware of the data. Watermarking is placing an invisible or visible mark inside an image or document. This technique is popular for proving the ownership of an image or document. Image encryption is the conversion of a raw image into a cipher image, which can be decrypted at the receiver end. It is a prominent technique to ensure data security, confidentiality, and integrity over the public network^1–3^.

Image encryption techniques are of two types. One is the classical encryption techniques like RSA, AES, and DES. Traditional encryption techniques have proven their significance on text data and have been widely used for web security and banking. However, due to the high computation time, such techniques are not suitable for digital images, which contain highly correlated data^4,5^. The other category of image encryption techniques includes the confusion and the diffusion step^6^. These techniques are popular for image encryption due to their low computation time and robustness against several attacks. Different authors have used various techniques in the confusion and diffusion steps. However, Chaos-based image encryption methods have gained attention due to chaos properties such as ergodicity, sensitivity to initial conditions and control parameters, random-like behavior, and unpredictability^7,8^. The performance of these methods depends upon the initial and the control parameters, which led to the need for optimal selection of these parameters for better performance of chaos-based encryption methods^9,10^.

Metaheuristic optimization techniques have been widely used for behavioral pattern guidance^11^, and optimal parameter selection. Metaheuristic techniques are of two major types, i.e., single objective and multi-objective metaheuristic techniques. Different single and multi-objective metaheuristic techniques like Particle Swarm Optimization (PSO)^12^, Bald Eagle Search (BES)^13^, multi-objective Brown Bear Optimization^14^, and multi-objective cheetah optimization^15^ algorithms have shown better performance in selecting the optimal parameters for different algorithms due to their high exploration and exploitation capabilities. This makes metaheuristic techniques suitable for selecting the initial and control parameters for the chaos-based image encryption methods. Various authors have used different metaheuristic techniques to select the initial and control parameters for chaos-based image encryption methods^16^.

Noshadian et al.^17^ have proposed an optimized image encryption technique based on Teacher Learning-Based Optimization (TLBO), Gravitational Search Algorithm (GSA), and logistic map. The authors have used a logistic map as an encryption key for diffusion and TLBO and GSA to optimize the map parameters. Farah et al.^6^ have proposed a new hybrid chaotic map for image encryption and generated a new substitution box using the Jaya algorithm. Saravanan and Sivabalakrishanan^18^ proposed an optimized hybrid chaotic map for image encryption. The authors have hybridized the 2DLCM and PWLCM map and performed parameter tuning using the improved whale optimization algorithm. The authors have analyzed the performance of their proposed algorithm on medical, natural, and satellite images. Kaur and Singh^19^ have used multiobjective evolutionary techniques to select the optimal parameters for the chaotic maps. The authors used the optimal parameters to generate a secret key, which is used to encrypt the image. They performed key, statistical, and differential analyses to analyze the performance of the encryption algorithm.

Luo et al.^20^ have used the hyperchaotic system and the updating process of particle swarm optimization for image encryption. They used the secure hash algorithm 256 to generate the initial keys for the hyperchaotic Lu system. The authors have analyzed the proposed algorithm using several attacks. Toktas and Erkan^21^ have designed a 2D fully chaotic map by utilizing the Artificial Bee Colony (ABC) for image encryption. The authors used ABC to minimize the quadruple objective function, which consists of the entropy, correlation coefficient, 0–1 test, and the Lyapunov exponent. Sameh et al.^16^ analyzed the impact of optimization of initial and control parameters for eight chaotic maps using nine metaheuristic optimization algorithms. Authors have computed the performance of encryption using sine, Tent, Circle, Gauss, singer, piecewise, and logistic maps without any optimization. Then, the authors used the Sine Cosine Algorithm (SCA), Moth Flame Optimization (MFO), Particle Swarm Optimization (PSO), Grey Wolf Optimization (GWO), Genetic Algorithm (GA), Dragonfly Algorithm (DA), Ant Lion Optimizer (ALO), Whale Optimization Algorithm (WOA), and Multi-Verse Optimizer (MVO) to select the optimal value of the chaotic map parameters. The authors have compared the performance to analyze the impact of each optimization algorithm.

Sharma et al.^22^ utilized the Self Adaptive Bald Eagle Search (SABES) optimization algorithm to optimize the chaotic parameters of PWLCM, PWCM, and tent maps. The authors used the random permutation method in the confusion phase and optimized chaotic maps in the diffusion phase with the cyclic redundancy check and circular shift method to secure patient medical information, medical signals as well as medical images. Sharma and Sharma^23^ have used the Harris Hawk Optimization (HHO) algorithm to optimize the Duffing, Lorenz, and Henon maps parameters. The authors have used different chaotic maps at different stages, which led to larger key spaces and resulted in a highly robust method.

### Novelty and contributions of the work

As mentioned earlier, different researchers have worked on image encryption using the chaotic map with optimized parameters using metaheuristic techniques. Still, to the best of our knowledge, a high-performance image encryption algorithm for different types of images consisting of hyperspectral images, grayscale, and RGB images is not available. This work designs a reliable image encryption algorithm based on a chaotic map optimized using the Iterative Cosine Operator (ICO) based Enhanced Hippopotamus Optimization (HO) algorithm. Overall, the main contributions of the paper are as follows:

i. Designed ICO-HO, i.e., ICO-based HO, by integrating a new phase (phase 4) for position update of Hippopotamus Optimization (HO) by utilizing ICO and opposition-based learning to enhance the exploration and exploitation capabilities of the algorithm.
ii. The proposed ICO-HO is used to design an adaptive image encryption method based on the chaotic maps, i.e., PWCM and PWLCM, with optimized parameters.
iii. Analysis of the proposed encryption method on different types of images, including medical images, grayscale images, RGB images, and hyperspectral images.

The remaining paper has been divided into four more sections. The next section, section ii, elaborates on the Hippopotamus optimization algorithm. The proposed work, which consists of the ICO-HO algorithm and the image encryption architecture, is explained in section iii. The results are analyzed and discussed in section iv. Section v concludes the work and describes the future scope.

## Understanding of Hippopotamus optimization

Hippopotamus optimization (HO)^24^ is inspired by the hippopotamus’s social behavior and defense process. Similar to the other population-based optimization algorithms, the Hippopotamus position is a candidate solution to the problem. The hippopotamus’s initial position is generated randomly, as given in Eq. (1).

1 $$\:{HO}_{ij}={L}_{j}+rand\times\:\left({U}_{j}-{L}_{j}\right)$$

Where $\:{HO}_{ij}$ denotes the position of i^th^ Hippopotamus in the j^th^ dimension. $\:{U}_{j},\:{L}_{j}$ denotes the upper and lower bounds for j^th^ dimension, respectively. $\:rand$ gives the random number between 0 and 1. Equation (2) gives the overall position matrix for M hippopotamus in the N dimension.

2 $$\:HO={\left[\begin{array}{ccc}{HO}_{\text{0,0}}&\:\begin{array}{cc}{HO}_{\text{0,1}}&\:\cdots\:\end{array}&\:{HO}_{0,N}\\\:\begin{array}{c}{HO}_{\text{1,0}}\\\:⋮\end{array}&\:\begin{array}{c}\begin{array}{cc}{HO}_{\text{1,1}}&\:\cdots\:\end{array}\\\:\begin{array}{cc}⋮&\:\cdots\:\end{array}\end{array}&\:\begin{array}{c}{HO}_{1,N}\\\:⋮\end{array}\\\:{HO}_{M,0}&\:\begin{array}{cc}{HO}_{M,1}&\:\cdots\:\end{array}&\:{HO}_{M,N}\end{array}\right]}_{M\times\:N}$$

The position update of the hippopotamus to explore the search space in the HO algorithm consists of three phases. The fitness value of each hippopotamus is computed using the fitness function $\:fit\left(\right)$. Phase 1 exhibits the exploration using the social behavior of the hippopotamus. The hippopotamus group consists of females, calves, males, and the leader hippopotamus, as given by Eq. (3).

3 $$\:HO={HO}^{f}\cup\:{HO}^{c}\cup\:{HO}^{m}\cup\:{HO}^{L}$$

where $\:{HO}^{f},\:{HO}^{c},{HO}^{m},{HO}^{L}$ represents the females, calves, males, and leader hippopotamus, respectively. Each hippopotamus is labeled to $\:{HO}^{f}or\:{HO}^{c}or\:{HO}^{m}or\:{HO}^{L}$ based on its fitness value only. The position update for the male hippopotamus inside the water bodies is given by Eq. (4).

4 $$\:\left.{HO}_{ij}^{m}={HO}_{ij}+rand\times\:\left({HO}^{L}-{C}_{1}{HO}_{ij}\right)\:\right|\:i=\:\text{1,2},3,\dots\:,\left[\raisebox{1ex}{$M$}\!\left/\:\!\raisebox{-1ex}{$2$}\right.\right]\:and\:j=\:\text{1,2},3,\dots\:.N\:$$

where $\:{C}_{1}$ is the constant integer between 1 and 2. The position update for the female hippopotamus and calves i.e., $\:{HO}^{fc}={H}^{f}\cup\:{H}^{c}$ is given by Eq. (5).

5 $$\:\left.{HO}_{ij}^{fc}=\:\begin{array}{c}{HO}_{ij}+{v}_{1}\times\:\left({HO}^{L}-{C}_{2}{RG}^{m}\right)\\\:\begin{array}{c}{HO}_{ij}+{v}_{2}\times\:\left({RG}^{m}-{HO}^{L}\right)\\\:{L}_{j}+rand\times\:\left({U}_{j}-{L}_{j}\right)\end{array}\end{array}\:\right|\:\begin{array}{c}T>0.6\\\:\begin{array}{c}else\:if\:rand>0.5\\\:else\end{array}\end{array}$$

where $$\:i=\:\text{1,2},3,\dots\:,\left[\raisebox{1ex}{$M$}\!\left/\:\!\raisebox{-1ex}{$2$}\right.\right]\:and\:j=\:\text{1,2},3,\dots\:.N$$. The $$\:{v}_{1},\:{v}_{2}$$ are generated using Eq. (6) and $$\:T$$ is generated using Eq. (7). $$\:{C}_{2}$$is the constant integer between 1 and 2. $$\:{RG}^{m}$$ is the mean of the randomly selected hippopotamus from the available $$\:M$$ hippopotamus.

6 $$\:v=\left\{\begin{array}{c}\begin{array}{c}{C}_{2}\times\:\overrightarrow{rand}+\left(\sim{\vartheta\:}_{1}\right)\\\:2\times\:\overrightarrow{rand}-1\end{array}\\\:\overrightarrow{rand}\\\:\begin{array}{c}{C}_{1}\times\:\overrightarrow{rand}+\left(\sim{\vartheta\:}_{2}\right)\\\:\overrightarrow{rand}\end{array}\end{array}\right.$$

7 $$\:T={e}^{-\raisebox{1ex}{$Cu{r}_{itr}$}\!\left/\:\!\raisebox{-1ex}{$Ma{x}_{itr}$}\right.}$$

where $\:Cu{r}_{itr}$ and $\:Ma{x}_{itr}$ is the current and maximum iteration, respectively. $\:{\vartheta\:}_{1},\:{\vartheta\:}_{2}\:$ are the random integers between 0 and 1. The updated position of hippopotamus is accepted only if it is better than the previous fitness value given by Eqs. (8) and (9).

8 $$\:{HO}_{i}=\left\{\left.\begin{array}{c}{HO}_{i}^{m}\\\:{HO}_{i}\end{array}\right|\begin{array}{c}fit\left({HO}_{i}^{m}\right)<fit\left({HO}_{i}\right)\\\:else\end{array}\right.$$

9 $$\:{HO}_{i}=\left\{\left.\begin{array}{c}{HO}_{i}^{fc}\\\:{HO}_{i}\end{array}\right|\begin{array}{c}fit\left({HO}_{i}^{fc}\right)<fit\left({HO}_{i}\right)\\\:else\end{array}\right.$$

where $\:fit\left(\right)$ is the fitness function. Phase 2 of the HO algorithms exhibits exploration and mimics the defense methodology of hippopotamus against predators. The position of the predator is given by the Eq. (10).

10 $$\:{P}_{j}={L}_{j}+rand\times\:\left({U}_{j}-{L}_{j}\right)\left|j=\:\text{1,2},3,\dots\:N\right.$$

The distance of a particular hippopotamus from the predator can be found using Eq. (11).

11 $$\:\overrightarrow{Dist}=\left|{P}_{j}-{HO}_{ij}\right|$$

The hippopotamus decides its defensive action based on the $\:\overrightarrow{Dist}$ value i.e., distance from the predator. If the hippopotamus is in close vicinity of the predator i.e., $\:fit\left({P}_{j}\right)<fit\left({HO}_{i}\right)$ then hippopotamus turns to face the predator otherwise it moves towards the predator as shown in Eq. (12).

12 $$\:{HO}_{ij}^{n}=\left.\begin{array}{c}{levy}^{r}\oplus\:{P}_{j}+\left(\frac{b}{\left(c-d*cos\left(2\pi\:g\right)\right)}\right)\cdot\:\left(\frac{1}{\overrightarrow{Dist}}\right)\\\:{levy}^{r}\oplus\:{P}_{j}+\left(\frac{b}{\left(c-d*cos\left(2\pi\:g\right)\right)}\right)\cdot\:\left(\frac{1}{2*\overrightarrow{Dist}+\overrightarrow{rand}}\right)\end{array}\right|\begin{array}{c}fit\left({P}_{j}\right)<fit\left({HO}_{i}\right)\\\:else\end{array}$$

where $$\:i=\:\left[\raisebox{1ex}{$M$}\!\left/\:\!\raisebox{-1ex}{$2$}\right.\right]+1,\left[\raisebox{1ex}{$M$}\!\left/\:\!\raisebox{-1ex}{$2$}\right.\right]+2,\cdots\:M\:and\:j=\:\text{1,2},3,\dots\:.N$$

The updated position of the hippopotamus is accepted only if its fitness value is better than the existing fitness value as given by Eq. (13).

13 $$\:{HO}_{i}=\left\{\left.\begin{array}{c}{HO}_{i}^{n}\\\:{HO}_{i}\end{array}\right|\begin{array}{c}fit\left({HO}_{i}^{n}\right)<fit\left({HO}_{i}\right)\\\:else\end{array}\right.$$

Phase 3 of the HO algorithm exhibits exploitation through the escaping behaviour of hippopotamus from the predator. Hippopotamus generally search for the nearest water bodies to escape from the predator. This phenomenon exhibits the exploitation search in the local region as hippopotamus explore the nearest water bodies. The local upper and lower bound for the current iteration can be found using the Eq. (14).

14 $$\:{U}_{j}^{local}=\raisebox{1ex}{${U}_{j}$}\!\left/\:\!\raisebox{-1ex}{$Cu{r}_{itr}$}\right.\:{L}_{j}^{local}=\raisebox{1ex}{${L}_{j}$}\!\left/\:\!\raisebox{-1ex}{$Cu{r}_{itr}$}\right.$$

The updated position of the hippopotamus is given by the Eq. (15).

15 $$\:{HO}_{ij}^{n}={HO}_{ij}+rand\left({L}_{j}^{local}+\alpha\:\left({U}_{j}^{local}-{L}_{j}^{local}\right)\right)$$

where $\:\alpha\:$ is given by the Eq. (16).

16 $$\:\alpha\:=\left\{\begin{array}{c}2\times\:\overrightarrow{rand}-1\\\:\overrightarrow{rand}\\\:\overrightarrow{randn}\end{array}\right.$$

where $\:\overrightarrow{randn}$ gives the random number with normal distribution. Hippopotamus will move to safer place only i.e., updated position is accepted only if its fitness value is better than the existing fitness value given by Eq. (17).

17 $$\:{HO}_{i}=\left\{\left.\begin{array}{c}{HO}_{i}^{n}\\\:{HO}_{i}\end{array}\right|\begin{array}{c}fit\left({HO}_{i}^{n}\right)<fit\left({HO}_{i}\right)\\\:else\end{array}\right.$$

The whole process i.e., three phases of the HO algorithm repeats for each candidate solution, for the $\:Ma{x}_{itr}$ iterations. HO algorithm is improved and utilized to optimize the parameters of the chaotic map discussed in the next section.

## Proposed work

This work proposes the ICO-HO, i.e., Iterative Cosine Operator-based Hippopotamus Optimization algorithm that adds a new phase to the HO algorithm for position updates using the ICO operator and opposition-based learning. The proposed ICO-HO is further used to optimize the initial and control parameters of chaotic maps. This work also proposes a security framework that uses the optimized chaotic maps in confusion and diffusion steps. Overall work is explained in two phases. The first phase defines the proposed ICO-HO, i.e., Iterative Cosine Operator-based Hippopotamus Optimization. The second phase describes the security framework for the image encryption approach based on ICO-HO.

### ICO-HO

ICO-HO improves the HO algorithm’s exploration and exploitation capabilities by using an Iterative Cosine Operator (ICO). ICO performs exploration at the initial iterations, which converts to the exploitation of search space as the iteration increases. Unlike the HO algorithm, which completes in three phases, ICO-HO completes its process in four phases. The first three phases of ICO-HO are the same as those of the HO algorithm, while the fourth phase updates the Hippopotamus position using Eq. (18).

18 $$\:{HO}_{i}^{n}=\left.\begin{array}{c}{HO}_{i}\times\:rand+{HO}^{L}\times\:cos\left(\raisebox{1ex}{$\left(\pi\:\times\:Cu{r}_{itr}\right)$}\!\left/\:\!\raisebox{-1ex}{$\left(2\times\:Ma{x}_{itr}\right)$}\right.\right)\\\:{HO}_{i}\times\:rand-{HO}^{L}\times\:cos\left(\raisebox{1ex}{$\left(\pi\:\times\:Cu{r}_{itr}\right)$}\!\left/\:\!\raisebox{-1ex}{$\left(2\times\:Ma{x}_{itr}\right)$}\right.\right)\end{array}\right|\begin{array}{c}rand<0.5\\\:else\end{array}$$

where, as presented in the previous section $$\:{HO}^{L}$$ is the position of leader hippopotamus. $$\:Cu{r}_{itr}\:,\:Ma{x}_{Itr}$$ are the current and maximum iterations, respectively. Equation (18) shows that the hippopotamus explores the search space toward the leader or opposite to the leader with a 50% probability of each case. This includes opposition-based leaning, as the optima may exist opposite the leader. This exploration at initial iteration converts to exploitation as the iteration increases due to the value of ICO i.e., $$\:cos\left(\raisebox{1ex}{$\left(\pi\:\times\:Cu{r}_{itr}\right)$}\!\left/\:\!\raisebox{-1ex}{$\left(2\times\:Ma{x}_{itr}\right)$}\right.\right)$$ approaching towards zero. The updated position value of the hippopotamus is accepted only if it gives a better fitness value as compared to the existing fitness value, as represented by Eq. (19).

19 $$\:{HO}_{i}=\left\{\left.\begin{array}{c}{HO}_{i}^{n}\\\:{HO}_{i}\end{array}\right|\begin{array}{c}fit\left({HO}_{i}^{n}\right)<fit\left({HO}_{i}\right)\\\:else\end{array}\right.$$

The whole process is repeated for the $\:Ma{x}_{Itr}$ times. The overall algorithm for ICO-HO is as follows.


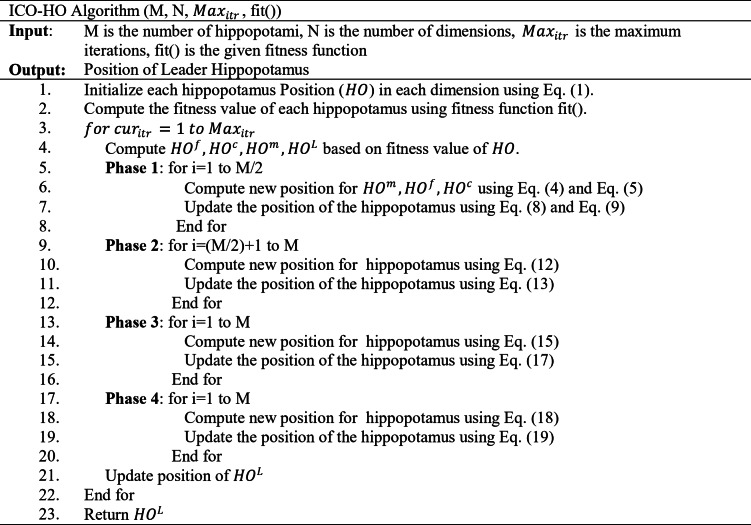


A framework to secure the image is designed using the ICO-HO algorithm explained in the next subsection.

### Proposed framework for image encryption approach

**Fig. 1:**
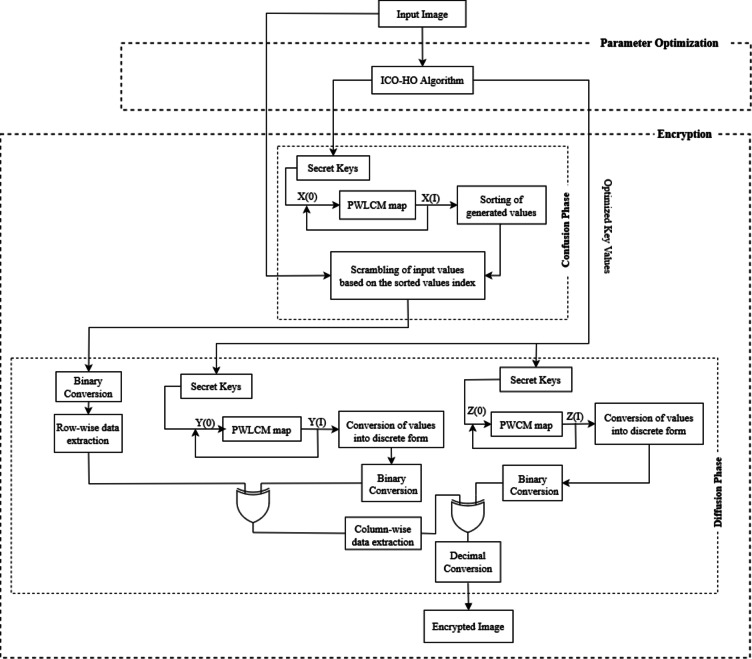
Framework for the image encryption.

This framework proposed for image encryption is demonstrated in Fig. 1. This framework uses chaotic maps to encrypt images and ICO-HO to select optimum parameters for chaoctic maps. The chaotic maps are selected for the encryption due to their properties: fast processing, determinism, aperiodic behavior, pseudo-randomness, boundedness, and dynamical nature. Encryption methods based on chaotic maps are also more robust because control parameters and initial conditions highly influence these maps. The complete process is divided into two stages i.e., the parameter optimization stage and the encryption stage. A description of each stage is given in the following subsections.

### Parameter optimization stage

In this stage, the proposed ICO-EHO has been used to solve the parameter optimization problem of the chaos-based encryption methods. The initial and the control parameters of chaotic maps are optimized using the ICO-EHO algorithm. In the proposed encryption method, two chaotic maps have been utilized: the Piecewise Linear Chaotic Map (PWLCM) and the Piecewise Chaotic Map (PWCM). The mathematical formulation for PWLCM and PWCM maps are presented in Eqs. (20) and (21) respectively.

20 $$\:{x}_{i}=F\left({x}_{i-1},{\eta\:}_{1}\right)=\left\{\begin{array}{c}\frac{{x}_{i-1}}{{\eta\:}_{1}},\:\:for\:\:0\le\:{x}_{i-1}<{\eta\:}_{1}\\\:\frac{{x}_{i-1}-{\eta\:}_{1}}{{0.5-\eta\:}_{1}},\:\:for\:\:{\eta\:}_{1}\le\:{x}_{i-1}<0.5\\\:0,\:\:\:\:\:\:\:\:\:\:\:for\:{x}_{i-1}=0.5\\\:F\left(1-{x}_{i-1},{\eta\:}_{1}\right),\:\:for\:0.5<{x}_{i-1}\le\:1\end{array}\right.$$

where the initial condition $\:{x}_{i}\in\:\left[\text{0,1}\right]$ and the control parameter $\:{\eta\:}_{1}\in\:\left[\text{0,0.5}\right]$ respectively

21 $$\:{y}_{i}\:=\left\{\begin{array}{c}\frac{\:\:{y}_{i-1}}{{\eta\:}_{2}}\:,\:\:\:\:\:\:\:\:\:\:\:\:\:\:\:\:\:\:for\:0\:<\:{y}_{i-1}\:<\:{\eta\:}_{2}\\\:\frac{\left({y}_{i-1}\:-\:{\eta\:}_{2}\right)}{\left(0.5\:-\:{\eta\:}_{2}\right)},\:\:for\:{\eta\:}_{2}\:\le\:\:{y}_{i-1}\:<\:0.5\\\:\frac{\left(1\:-\:{\eta\:}_{2}\:-\:{y}_{i-1}\right)}{\left(0.5-{\eta\:}_{2}\right)},\:for\:0.5<{y}_{i-1}<\left(1-{\eta\:}_{2}\right)\\\:\frac{\left(1\:-\:{y}_{i-1}\right)}{{\eta\:}_{2}},for\left(1-{\eta\:}_{2}\right)<{y}_{i-1}<1\end{array}\right.$$

where the PWCM map parameters $\:{\eta\:}_{2}$ and $\:{y}_{i}$ are defined as $\:{\eta\:}_{2}\in\:\left[\text{0,0.5}\right]$ and $\:{y}_{i}\in\:\left[\text{0,1}\right]$, respectively. The parameters of both chaotic maps i.e., $\:{{{x}_{i}\:,y}_{i},\eta\:}_{1},\:{and\:\eta\:}_{2}$ are optimized using the ICO-EHO algorithm.

**Fig. 2:**
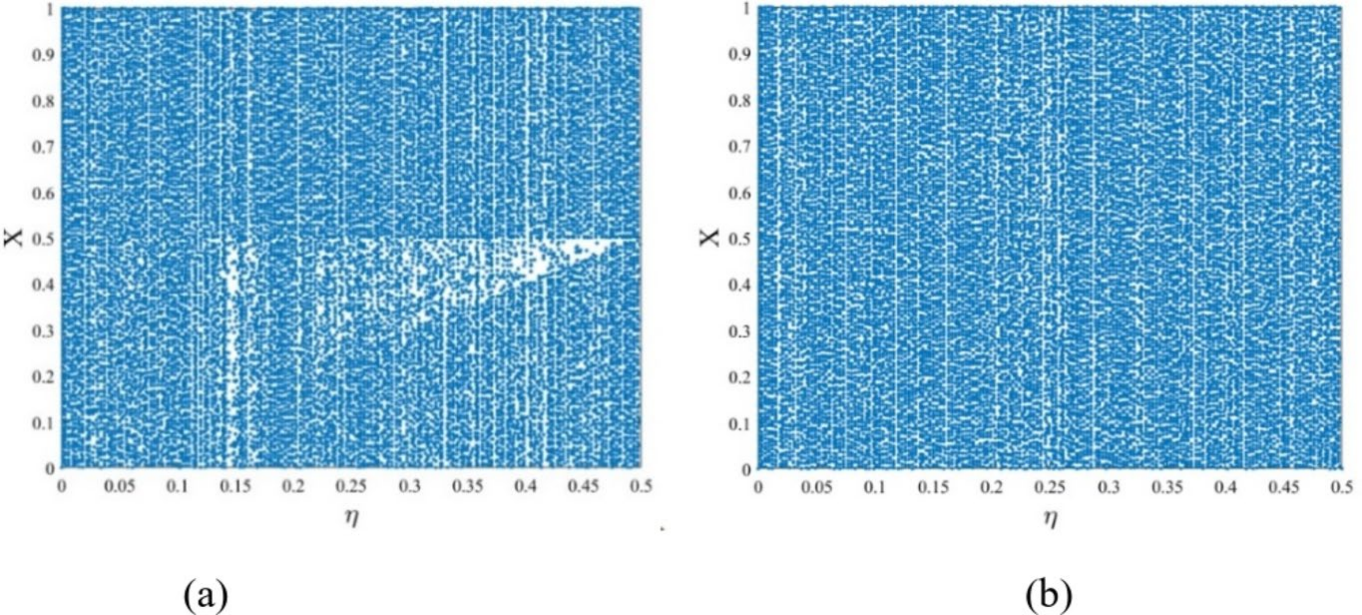
Bifurcation diagram (**a**) PWLCM map (**b**) PWCM map.

A bifurcation diagram of the chaotic maps shows the dynamic change in the behavior of the maps in terms of the control parameters. This diagram is used to analyze changes in the chaotic sequence in the whole definition of the control parameters. In Fig. 2, bifurcation diagrams of the PWLCM and PWCM maps are shown for the control parameter $\:\eta\:ϵ[0,\:0.5]$ and initial parameter $\:Xϵ\left[\text{0,1}\right]$.

The bifurcation diagram shows that both maps exhibit chaotic behavior across the entire range of the control and initial parameters. In Fig. 2(a), PWLCM maps show the period-doubling cascade behavior, characterized by two bifurcation divisions. In contrast, the PWCM map presented in Fig. 2(b) shows a smoother and more continuous change in the behavior as control parameters vary, reflecting its abrupt and less predictable transitions.

**Fig. 3:**
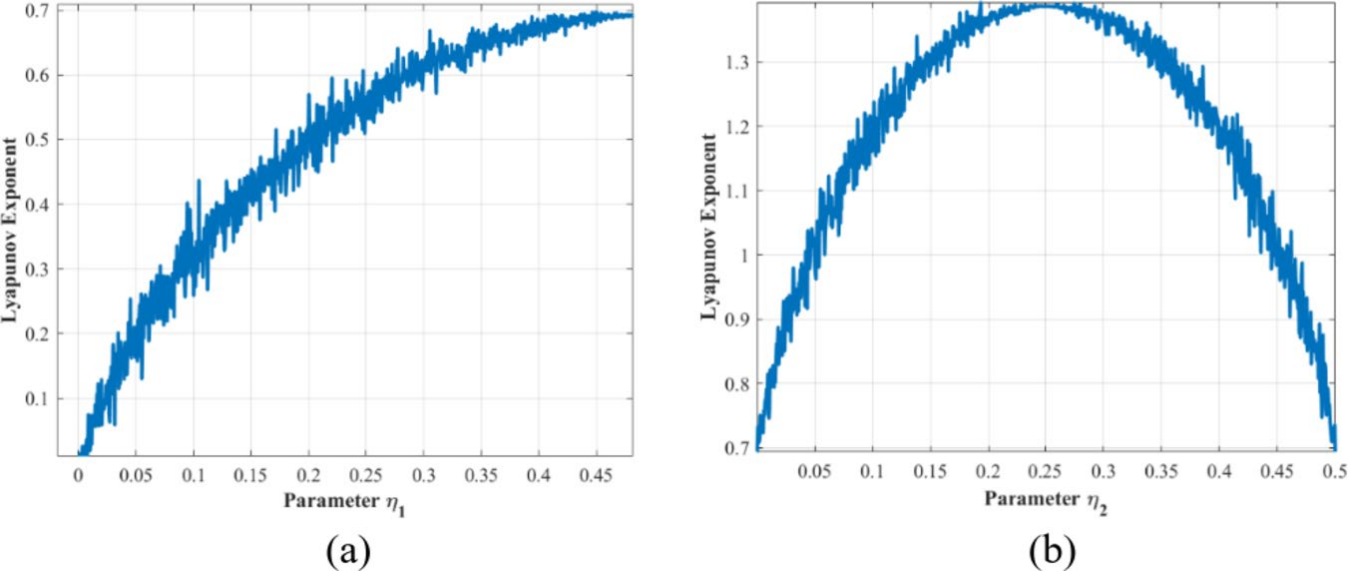
Lyapunov exponent (**a**) PWLCM (**b**) PWCM.

Lyapunov Exponent (LE) is used as a quantitative measure to analyze the perturbations in time series data. The positive value of the Lyapunov exponent reflects that neighboring trajectories are diverged with each other, showing instability within the time series. The negative values show that the neighboring trajectories converge to a single point, representing a stable trajectory. For the chaotic maps, the value of the Lyapunov exponent greater than 0 indicates that the map is reflecting the chaotic behavior. The change in the Lyapunov exponent values based on the control parameter is shown in Fig. 3.

Figure 3 indicates that the Lyapunov exponent of PWLCM maps is consistently greater than 0, indicating that the maps exhibit chaotic behavior in the entire range of control parameters. The LE values of the PWCM are higher than the PWLCM map values. Also, the PWCM map reaches its highest LE value at $\:{\eta\:}_{2}=0.25$ after that, LE values decline but remain above 0.

### Encryption stage

After optimizing the parameters of the chaotic maps, those parameters are utilized in the encryption process to enhance the security of the images. The encryption phase is divided into two phases: Confusion and diffusion phase. The confusion phase is responsible for breaking the correlation between the image pixel values by shuffling or scrambling the image pixel values. In the confusion phase, an optimized PWLCM map was used to shuffle the image pixel values. The map values have been generated with a size equal to the image size using the optimal parameters. Thereafter, the generated values of the map are sorted to give the scrambled indexes. The original image is rearranged using the scrambled index generated through sorted chaotic map values. The pseudocode of the confusion process is as follows:


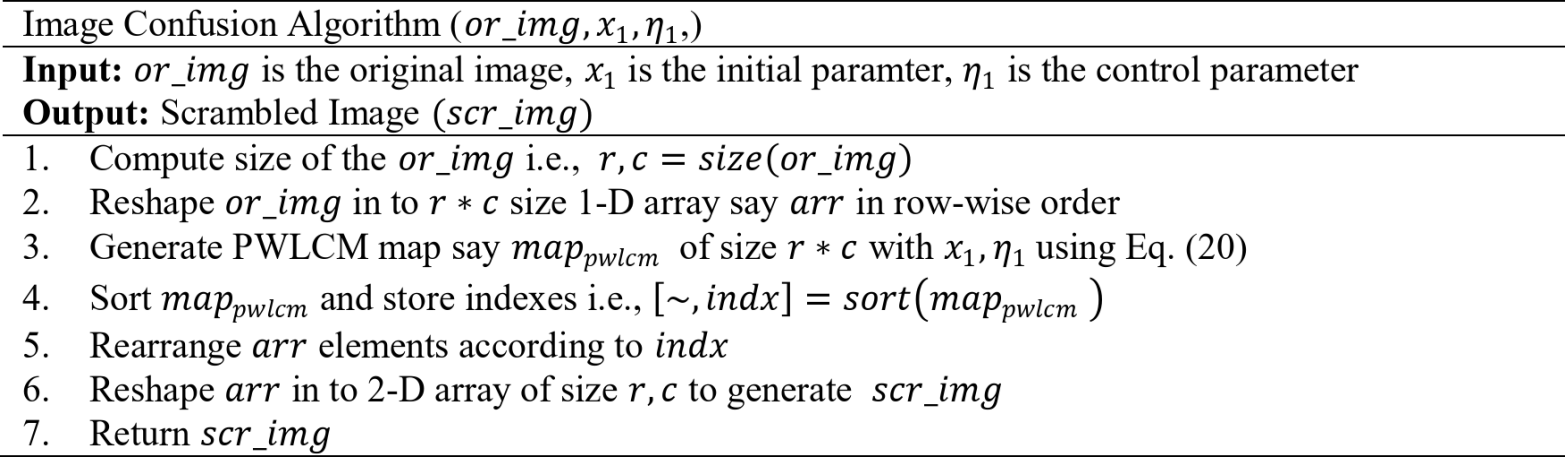


After the confusion phase, the diffusion phase has been applied to the scramble image $\:(scr\_img)$ pixels values. The optimized PWLCM and PWCM maps have been applied to modify the image pixel values in both row-wise and column-wise order. Firstly, the values of the chaotic map are generated using the optimized parameters, and then generated values are changed into discrete form using Eq. (22).

22 $$\:value=⌊\left({value}_{i}\times\:{10}^{14}\right)⌋\%\left(max\left(inputvalue\right)\right)$$

where $\:max\left(inputvalue\right)$, is the maximum value of the input image. The pseudocode of the diffusion phase is as follows:


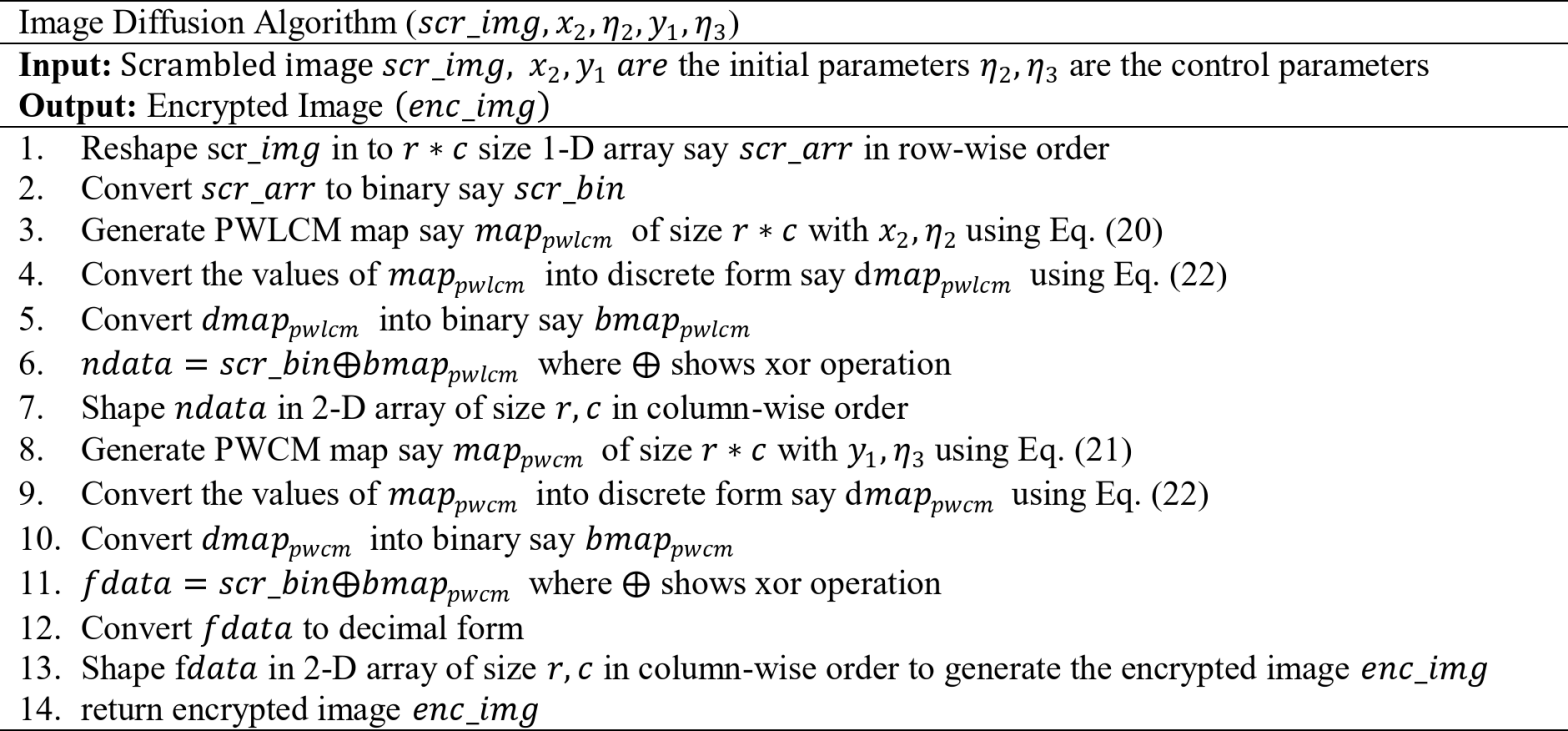


The analysis of the work has been done in the next section.

## Results and analysis

The results and corresponding discussions have been explained in two subsections. Subsection 1, i.e., analysis of the ICO-HO algorithm, analyses the performance of the ICO-HO algorithm on CEC-2017 functions. The Friedman mean rank, analysis using qualitative metrics, and the convergence curve comparison are used for the performance analysis of ICO-HO. Subsection 2, i.e., analysis of the image encryption approach, comprises different attack analyses, key space analysis, and the comparison against different state-of-the-art techniques.

### Analysis of ICO-HO algorithm

The performance of ICO-HO is compared with seven state-of-the-art algorithms namely HO^24^, WOA^25^, Arithmetic Optimization Algorithm (AOA)^26^, SCA^27^, MFO^28^, African Vultures Optimization Algorithm (AVOA)^29^, and RIME optimization algorithm (RIME)^30^ on CEC-2017 functions. The analysis has been done by comparing the best, average, and worst values and the Standard Deviation (SD), as shown in Table 1.

**Table 1:** Performance analysis of ICO-HO on CEC-2017 functions.

Average		ICO-HO	HO	WOA	AOA	SCA	MFO	AVOA	RIME
C17_F1	Average	10032.05	19645.82	2,950,103	8.62E + 09	8.68E + 08	1.34E + 08	3183.948	6144.983
	SD	11748.67	33930.79	5,027,612	3.49E + 09	3.4E + 08	3.99E + 08	3351.859	3663.503
	Best	572.0408	673.912	81256.52	4.07E + 09	3.54E + 08	206.7003	100.1482	1256.231
	Worst	42625.33	132512.2	19,659,938	1.75E + 10	1.86E + 09	1.6E + 09	12738.8	14244.02
	Rank	3	4	5	8	7	6	1	2
C17_F3	Average	308.1496	397.8387	1669.347	11145.24	2030.393	6368.661	311.5693	308.1837
	SD	43.07663	100.4892	1271.698	2463.444	556.1126	7173.071	19.42854	0.128031
	Best	302.7104	300.9601	409.6693	6793.901	1380.234	300	300	300.0295
	Worst	441.7912	665.4157	5023.522	15496.14	3549.512	23556.71	369.2791	300.4607
	Rank	1	4	5	8	6	7	3	2
C17_F4	Average	408.5707	434.6272	437.8194	1117.241	450.2514	416.2374	412.6407	412.5813
	SD	32.07912	37.68755	48.26765	594.7307	21.49669	20.6758	22.35402	22.21465
	Best	400.257	400.1631	400.3183	517.9173	423.4422	403.6003	400.0578	400.8401
	Worst	499.4128	512.8749	569.4064	2833.728	496.9436	475.2809	480.5764	491.1705
	Rank	1	5	6	8	7	4	3	2
C17_F5	Average	530.2469	538.4561	551.5749	559.4188	553.329	527.2206	537.8326	518.1173
	SD	11.90459	17.93958	22.63705	23.76553	5.137278	11.65595	19.01879	7.140284
	Best	520.8945	513.9295	519.9687	527.9753	542.8197	505.9698	509.9496	501.9937
	Worst	566.6622	572.6324	615.5233	618.4109	562.129	547.0116	590.5407	532.8361
	Rank	3	5	6	8	7	2	4	1
C17_F6	Average	604.8605	623.6262	630.6644	642.7526	622.2771	601.6499	616.8276	605.1076
	SD	9.530594	10.29718	12.10532	6.714907	5.391947	4.349247	11.10042	0.053136
	Best	600.8532	611.1294	610.0029	633.1757	611.0247	600	601.7968	600.0445
	Worst	644.2599	652.4915	654.9993	660.0947	632.0914	617.7367	642.6253	600.2505
	Rank	2	6	7	8	5	1	4	3
C17_F7	Average	761.9636	762.1628	784.2739	800.3603	779.2819	735.7803	764.2323	755.76
	SD	13.92278	17.15086	28.77084	13.6912	8.909798	10.21436	19.55945	6.003813
	Best	740.2605	726.0081	734.2367	769.0804	761.1076	720.045	727.8699	712.0166
	Worst	790.3632	784.1074	856.7039	823.7258	799.6738	765.2244	796.9996	734.5806
	Rank	3	4	7	8	6	1	5	2
C17_F8	Average	821.293	822.241	842.0626	831.2885	845.2505	828.0353	833.623	819.4559
	SD	4.27885	4.805375	17.15654	8.364541	7.867443	14.4076	11.5436	7.90988
	Best	813.9301	809.9499	814.0009	816.9623	826.0757	805.9697	816.9143	805.9713
	Worst	832.8338	828.8542	891.6088	848.6541	857.9848	860.9836	862.552	837.8143
	Rank	2	3	7	5	8	4	6	1
C17_F9	Average	1066.82	1154.74	1449.127	1393.627	1037.89	948.6505	1209.757	900.3799
	SD	133.4051	136.9636	383.0117	159.4001	54.16431	140.734	259.646	1.2124
	Best	918.5424	932.9554	957.8474	1140.097	954.9829	900	925.5092	900.0011
	Worst	1344.605	1405.395	2588.32	1626.386	1218.797	1661.86	1826.779	905.4755
	Rank	4	5	8	7	3	2	6	1
C17_F10	Average	1735.444	1909.224	2117.373	2170.142	2403.684	1836.051	2011.75	1483.618
	SD	133.3492	205.5764	334.8568	320.0934	256.6815	328.7047	294.3816	213.1922
	Best	1644.607	1518.602	1323.805	1638.399	1781.716	1151.821	1426.475	1010.33
	Worst	2093.052	2247.228	2643.931	2625.874	2720.402	2367.483	2514.838	1895.653
	Rank	2	4	6	7	8	3	5	1
C17_F11	Average	1111.189	1173.981	1215.654	3398.495	1234.822	1211.334	1146.053	1113.785
	SD	31.2681	49.79625	85.13215	3616.072	49.56309	336.4117	27.66517	7.876421
	Best	1104.848	1110.373	1118.888	1188.99	1157.478	1102.133	1110.093	1105.825
	Worst	1258.318	1309.789	1467.571	11190.95	1332.295	2966.228	1236.842	1130.967
	Rank	1	4	6	8	7	5	3	2
C17_F12	Average	55248.89	903454.9	3,878,271	1.49E + 08	20,146,718	3,131,227	1,204,554	35273.48
	SD	102,692	1,417,342	5,794,507	3.41E + 08	18,153,246	4,630,822	1,108,377	29138.85
	Best	3272.805	2769.581	17055.68	30084.76	5,278,934	2035.524	7905.968	4318.387
	Worst	376094.8	6,230,518	21,028,645	1.55E + 09	66,697,549	17,095,033	3,454,601	121156.7
	Rank	2	3	6	8	7	5	4	1
C17_F13	Average	1893.149	3135.278	19411.16	12332.75	57897.41	14986.92	16719.58	9597.665
	SD	538.6321	1443.776	9356.39	7766.325	51370.72	11551.48	10634.37	9099.895
	Best	1411.424	1520.73	6184.687	3649.982	5604.004	1536.661	3316.809	1317.108
	Worst	3650.611	6658.623	45487.85	28151.86	200434.1	34803.86	34994.21	29209.92
	Rank	1	2	7	4	8	5	6	3
C17_F14	Average	1472.299	1502.367	1860.926	10077.41	2154.41	3446.668	1872.674	4578.689
	SD	19.74287	25.23012	897.7745	8705.482	1104.695	2773.087	617.4258	4322.276
	Best	1441.584	1466.149	1458.029	1488.821	1508.383	1448.236	1463.359	1404.102
	Worst	1515.773	1558.612	5196.362	26797.22	5371.562	13189.37	3416.18	17214.08
	Rank	1	2	3	8	5	6	4	7
C17_F15	Average	2123.374	2848.772	6472.51	16912.16	3687.485	6873.928	4600.647	4960.371
	SD	653.0414	1087.589	4065.96	5078.372	2439.629	7563.927	1758.751	3855.296
	Best	1682.612	1664.072	1921.679	3447.759	1614.153	1615.894	1809.69	1532.311
	Worst	4508.466	5155.553	17475.45	21729.13	11976.26	32516.18	8304.92	17848.4
	Rank	1	2	6	8	3	7	4	5
C17_F16	Average	1797.229	1826.87	1866.867	2045.021	1764.008	1753.562	1860.764	1757.983
	SD	100.2903	94.18259	151.6341	144.7012	63.24585	102.7175	141.7337	109.0694
	Best	1605.299	1694.855	1633.389	1747.342	1655.376	1613.253	1622.768	1611.852
	Worst	2002.308	2067.148	2188.81	2327.506	1881.417	1991.643	2146.174	1975.509
	Rank	4	5	7	8	3	1	6	2
C17_F17	Average	1757.445	1760.713	1791.802	1920.994	1790.669	1784.881	1784.096	1767.784
	SD	11.21124	10.22898	47.0718	113.8063	12.81915	63.75041	40.71103	49.83215
	Best	1735.165	1747.446	1742.443	1789.989	1770.568	1703.571	1731.536	1703.743
	Worst	1778.158	1781.754	1937.894	2117.084	1818.679	1908.636	1874.717	1897.723
	Rank	1	2	7	8	6	5	4	3
C17_F18	Average	1917.528	2099.678	16294.8	19061.25	346523.9	23473.67	16915.03	8805.213
	SD	51.35381	555.8699	10731.74	12624.2	318249.3	12808.68	9639.937	7255.108
	Best	1856.165	1839.14	2082.594	2361.728	55589.23	3930.069	2860.699	1848.471
	Worst	2050.587	4357.48	48869.53	47197.27	1,189,112	46287.21	35681.38	27334.25
	Rank	1	2	4	6	8	7	5	3
C17_F19	Average	2556.519	3442.259	21623.47	63908.73	9090.571	10291.59	8282.266	6143.657
	SD	918.616	4069.32	23740.47	44226.47	5952.071	10904.96	8444.079	5620.29
	Best	1923.626	1921.763	2521.89	2066.882	2169.212	1985.685	1921.244	1904.914
	Worst	4682.196	17924.68	100,946	158100.5	17871.93	32938.84	29124.01	19611.75
	Rank	1	2	7	8	5	6	4	3
C17_F20	Average	2107.785	2136.049	2169.988	2187.979	2115.931	2092.709	2130.946	2141.452
	SD	60.39016	53.68585	72.50514	55.09885	35.08344	66.03487	55.53927	53.09002
	Best	2037.472	2048.704	2058.489	2045.916	2078.449	2001.307	2038.715	2000.191
	Worst	2216.556	2214.54	2361.341	2305.49	2214.698	2281.444	2219.053	2162.184
	Rank	2	5	7	8	3	1	4	6
C17_F21	Average	2242.999	2260.74	2310.927	2332.03	2301.675	2304.044	2291.535	2315.283
	SD	62.22632	66.50344	64.3621	36.8606	67.31293	52.25288	69.84209	28.10286
	Best	2203.951	2202.595	2207.133	2218.834	2206.455	2200	2201.976	2202.149
	Worst	2345.716	2350.481	2390.386	2372.89	2355.37	2357.671	2377.025	2343.591
	Rank	1	2	6	8	4	5	3	7
C17_F22	Average	2310.781	2314.001	2340.898	2927.926	2377.175	2307.627	2348.669	2302.975
	SD	21.14068	6.920783	126.2987	299.0046	60.38978	14.2347	198.8712	1.690643
	Best	2227.465	2304.611	2305.552	2365.592	2280.407	2300.398	2241.799	2301.233
	Worst	2330.459	2332.357	3008.196	3742.582	2524.45	2351.221	3191.049	2307.212
	Rank	3	4	5	8	7	2	6	1
C17_F23	Average	2633.2	2638.227	2645.988	2740.437	2661.824	2626.354	2644.558	2619.842
	SD	11.26014	14.65185	22.49151	43.04086	9.439614	9.77881	16.92285	8.173376
	Best	2614.019	2609.214	2617.377	2682.19	2648.372	2610.726	2609.139	2605.176
	Worst	2651.938	2673	2702.175	2824.289	2681.176	2645.491	2686.508	2635.974
	Rank	3	4	6	8	7	2	5	1
C17_F24	Average	2502.569	2612.545	2766.578	2895.324	2780.833	2760.748	2763.127	2725.642
	SD	26.13108	109.7154	70.74965	75.40721	50.71567	11.3152	65.249	96.13567
	Best	2500.023	2500.051	2505.189	2743.267	2567.533	2743.606	2500	2401.398
	Worst	2614.987	2809.125	2834.145	2992.625	2801.272	2788.586	2819.376	2779.353
	Rank	1	2	6	8	7	4	5	3
C17_F25	Average	2931.315	2928.88	2939.051	3324.418	2967.703	2934.63	2934.965	2927.841
	SD	24.64206	26.26078	55.46868	189.6134	14.69449	24.62887	24.12026	24.36049
	Best	2898.005	2898.401	2674.756	3076.171	2948.477	2898.318	2898.47	2898.272
	Worst	2955.727	2969.908	3029.894	3797.308	3015.477	2972.644	2972.24	2953.043
	Rank	3	2	6	8	7	4	5	1
C17_F26	Average	2946.9	2967.849	3491.274	4052.892	3104.902	2989.406	3201.352	2980.129
	SD	292.0065	171.8731	525.3182	346.7445	48.25863	54.18153	495.4758	229.8812
	Best	2600.259	2600.421	2817.982	3256.685	3032.253	2800	2600	2802.295
	Worst	3961.089	3224.237	4407.501	4593.257	3218.55	3112.164	4360.15	3909.271
	Rank	1	2	7	8	5	4	6	3
C17_F27	Average	3100.369	3123.31	3130.405	3249.985	3105.208	3093.444	3100.507	3103.93
	SD	24.94538	37.81637	32.70263	54.41311	2.232078	2.358467	9.08241	24.2242
	Best	3091.257	3092.511	3090.425	3186.108	3101.815	3089.297	3092.217	3089.374
	Worst	3183.637	3216.724	3202.673	3377.093	3110.625	3098.501	3130.711	3192.888
	Rank	2	6	7	8	5	1	3	4
C17_F28	Average	3276.112	3338.84	3376.678	3804.762	3338.961	3336.497	3328.576	3240.441
	SD	107.8746	102.7604	163.1624	177.7299	85.76557	83.72238	132.6038	132.9288
	Best	3100.041	3173.365	3115.721	3506.761	3245.936	3196.836	3100	3100.184
	Worst	3419.422	3571.698	3749.371	4255.533	3453.316	3457.998	3411.822	3411.822
	Rank	2	5	7	8	6	4	3	1
C17_F29	Average	3207.364	3272.969	3346.013	3394.744	3236.345	3235.99	3294.483	3202.466
	SD	59.23586	67.89653	111.1383	131.1819	27.03826	50.90261	98.78095	36.58038
	Best	3148.484	3177.548	3192.108	3184.759	3206.952	3163.257	3155.898	3148.332
	Worst	3383.994	3413.839	3807.624	3699.993	3324.49	3338.936	3487.477	3288.074
	Rank	2	5	7	8	4	3	6	1
C17_F30	Average	240030.5	1,200,690	866,758	34,466,452	1,528,433	594299.7	491331.6	172,600
	SD	259345.3	1,107,869	1,353,645	38,682,718	942762.4	535968.1	586906.5	301516.4
	Best	5697.543	5888.888	15541.3	567971.3	350202.7	7321.186	12103.88	8822.405
	Worst	865978.6	3,639,497	6,601,609	1.34E + 08	4,111,892	1,941,753	1,683,573	879893.1
	Rank	2	6	5	8	7	4	3	1
Friedman mean rank		1.931034	3.689655	6.172414	7.62069	5.896552	3.827586	4.344828	2.517241
Rank		1	3	7	8	6	4	5	2

Table 1 shows the performance comparison of ICO-HO with seven state-of-the-art techniques using average, best, worst, and standard deviation values. The rank for each function is assigned for every algorithm based on the average value. It can be easily analyzed that ICO-HO achieved the first rank for twelve functions, while ICO-HO gives a competitive solution for the remaining functions. Friedman mean rank analysis is applied to analyze the performance of each algorithm, which results in 1.93, 3.68, 6.17, 7.62, 5.89, 3.82, 4.34, and 2.51 values for ICO-HO, HO, WOA, AOA, SCA, MFO, AVOA, RIME algorithms respectively. The p-value for the Friedman mean rank analysis is 3.56E-24, which is less than 0.05, resulting in the rejection of the NULL hypothesis, i.e., there is a significant difference between the performance of algorithms. On the basis of the mean value obtained, ICO-HO has obtained the first position, indicating better performance than the other existing state-of-the-art algorithms. ICO_HO has shown better performance due to its high and balanced exploration and exploitation capabilities. RIME and HO algorithms have obtained the second and third positions, respectively.

**Fig. 4:**
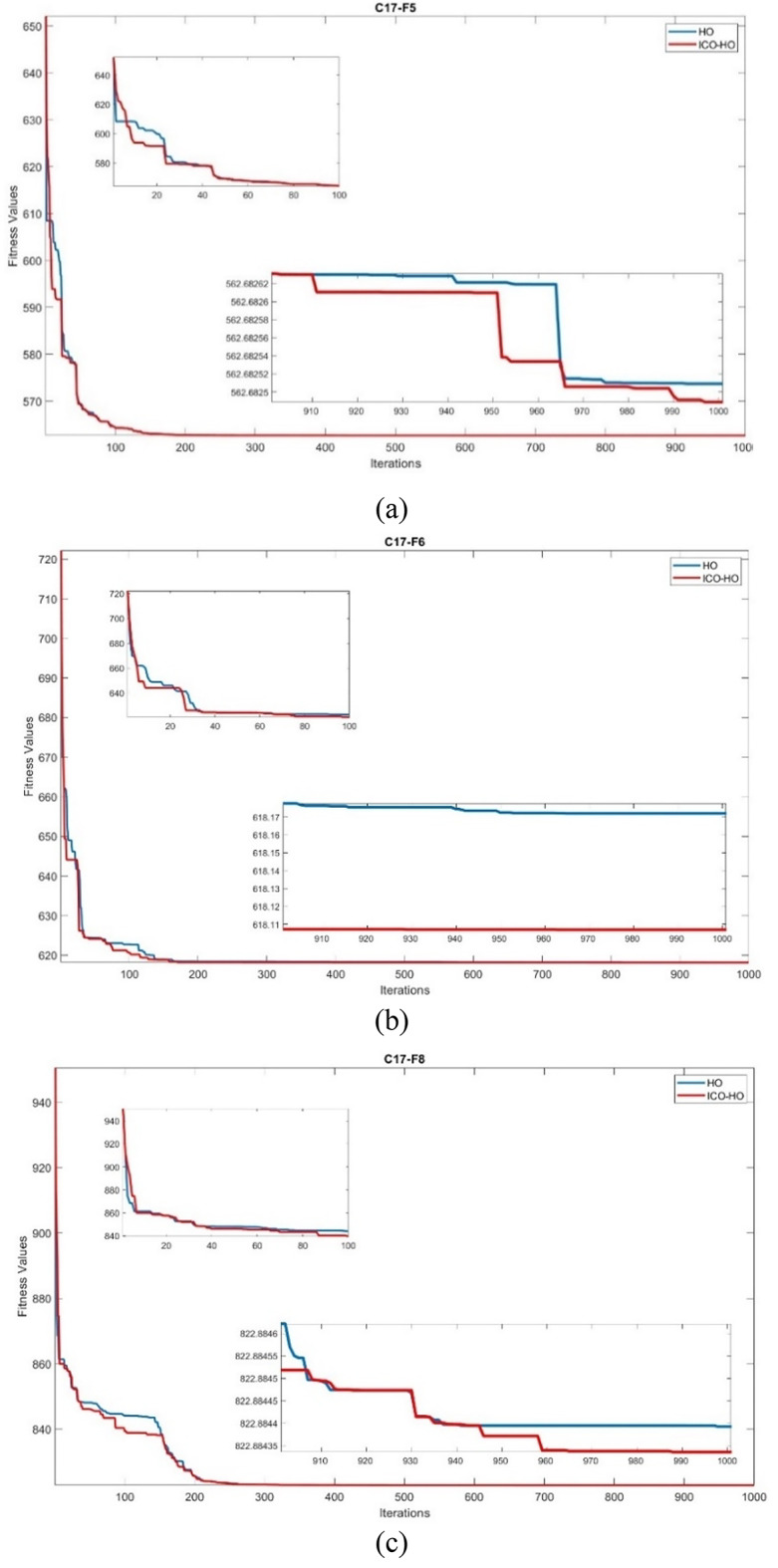

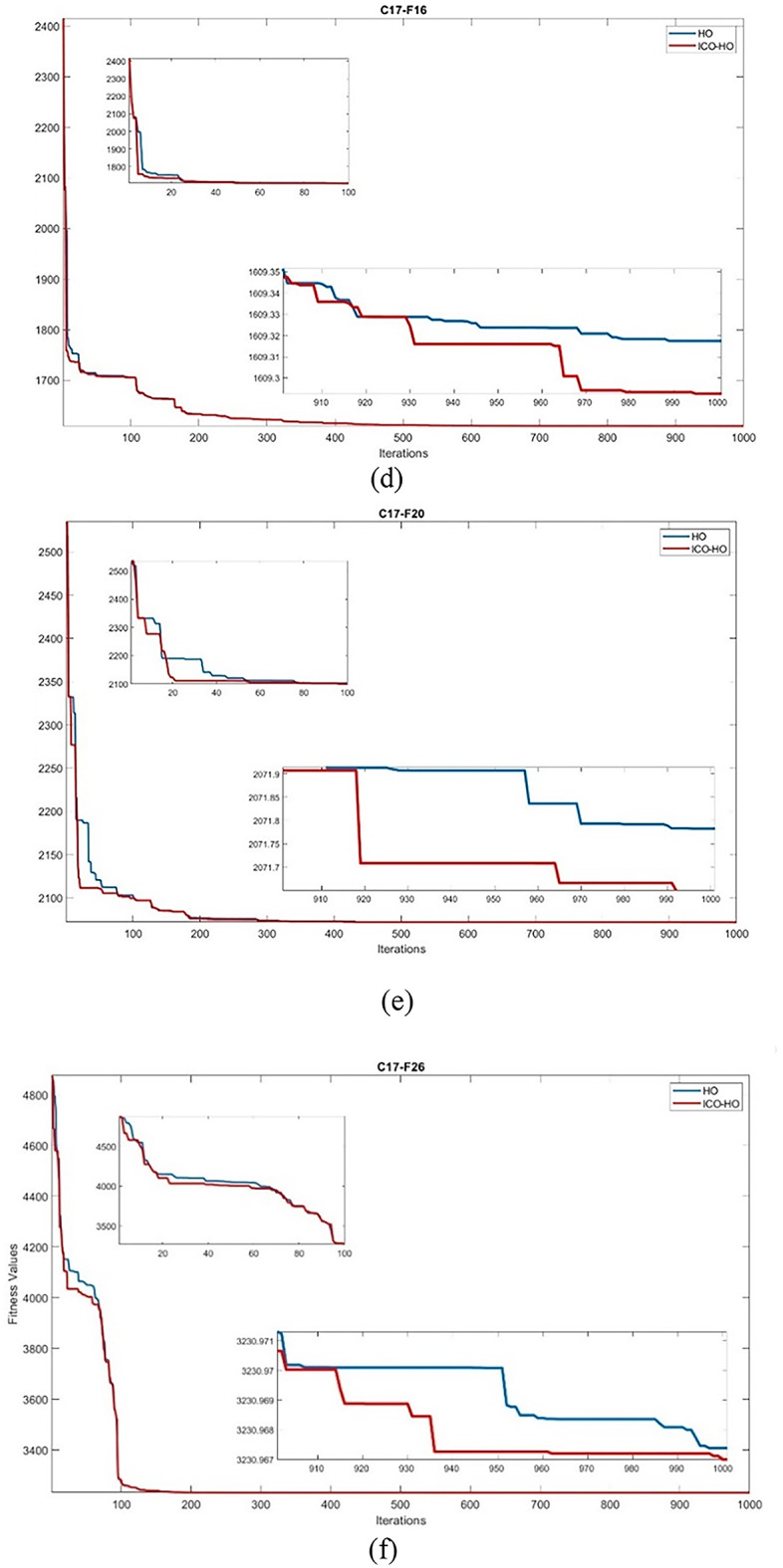
Convergence comparison of ICO-HO with HO algorithm on (**a**) F5 (**b**) F6 (**c**) F8 (**d**) F16 (**e**) F20 (**f**) F26 functions.

### Convergence comparison of ICO-HO with HO algorithm

The comparison of the convergence curve for ICO-HO with the HO algorithm is shown on the randomly selected CEC-2017 functions, i.e., F5, F6, F8, F16, F20, and F26 in Fig. 4. The x-axis denotes the iterations, while the y-axis denotes the fitness value. A comparison was made with the 1000 iterations. The better convergence is exhibited by the ICO-HO as compared to the HO algorithm for all F5, F6, F8, F16, F20, and F26 functions. The better convergence is due to the newly added phase 4, which includes the position update based on ICO and opposition-based learning. The better exploration capabilities due to ICO at initial iterations in the ICO-HO show faster convergence by ICO-HO than the HO algorithm.

### Analysis of proposed image encryption approach

The proposed encryption approach is tested on different types of medical images, including grayscale, RGB, and hyperspectral images. The medical images used for experimentation vary in size, file format, and bit depth, including both 8-bit and 16-bit images. The performance analysis has been done using visual analysis, statistical attack analysis (histogram, correlation analysis, variance analysis, chi-square analysis), differential attack analysis (NPCR and UACI), qualitative analysis (information entropy, MSE, and PSNR), key space and key sensitivity analysis. The visual analysis has been utilized to show the visual difference between the original, encrypted, and decrypted medical images^31^. Table 2 depicts the visual analysis of original, encrypted, and decrypted medical images.

**Table 2:** Visual depiction of original, encrypted, and decrypted images.

Image name/type/size	Original image	Encrypted image	Decrypted image
MRI image^32,33^ Grayscale $\:630\times\:630$			
Ultrasound image^34^ Grayscale $\:471\times\:562$			
Diabetic retinopathy image^35^ RGB $\:565\times\:584\times\:3$			
Skin image^36^ RGB $\:1064\:\times\:1736\:\times\:3$			
Human brain tissue image^37^ Hyperspectral $\:400\times\:582\times\:826$			
Cholangiocarcinoma image^35^ Hyperspectral $\:1280\times\:1024\times\:60$			

The encrypted images in Table 2 clearly show that the visual information of the original images is completely hidden in the encrypted images generated using the propounded encryption method. This proves that the proposed encryption method effectively hides all the visual data of the images. Also, the visual appearance of the decrypted image is identical to the original image, indicating that the decrypted image maintains the same visual depiction as the original.

### Statistical attack analysis

**Fig. 5:**
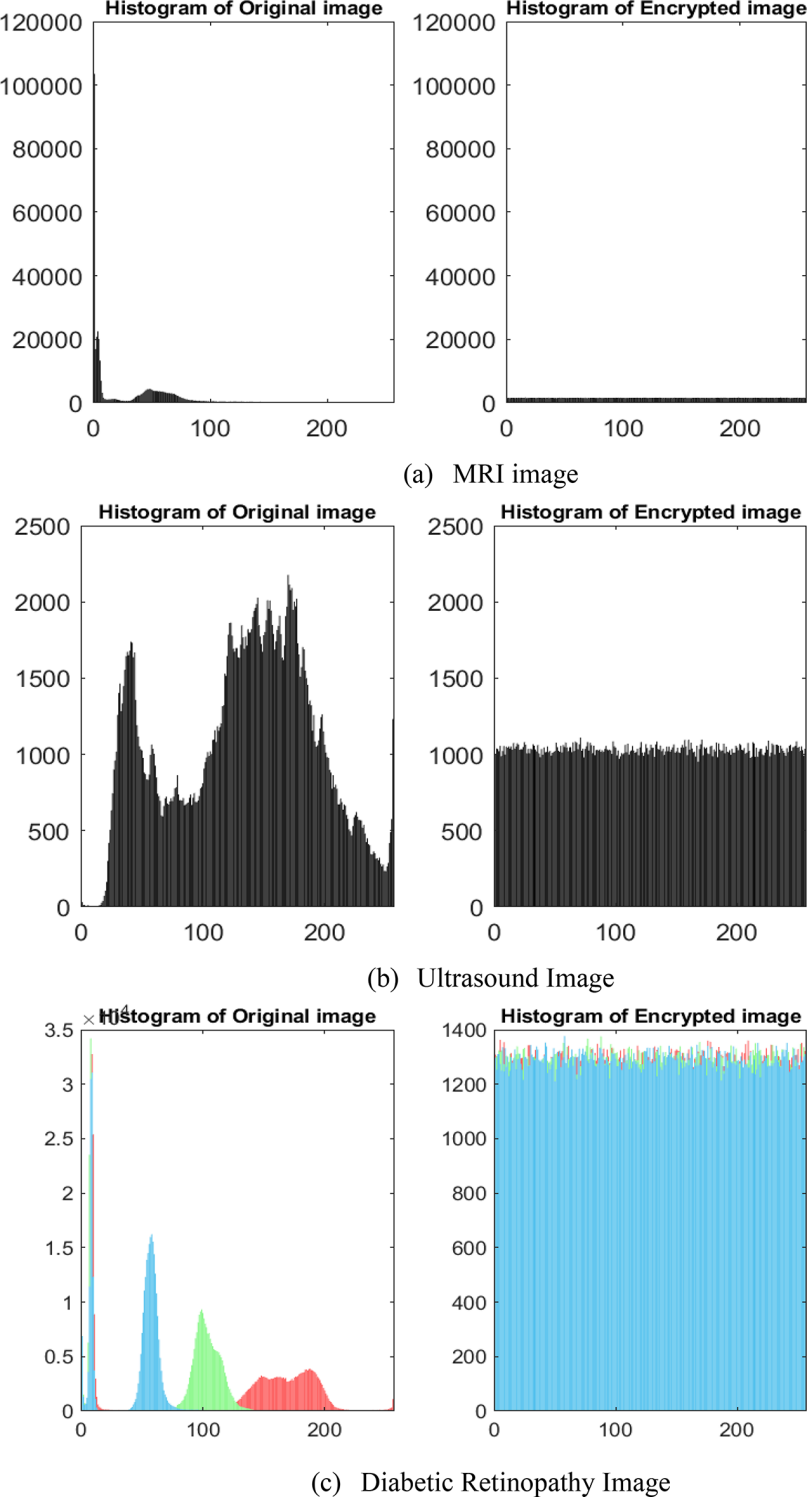

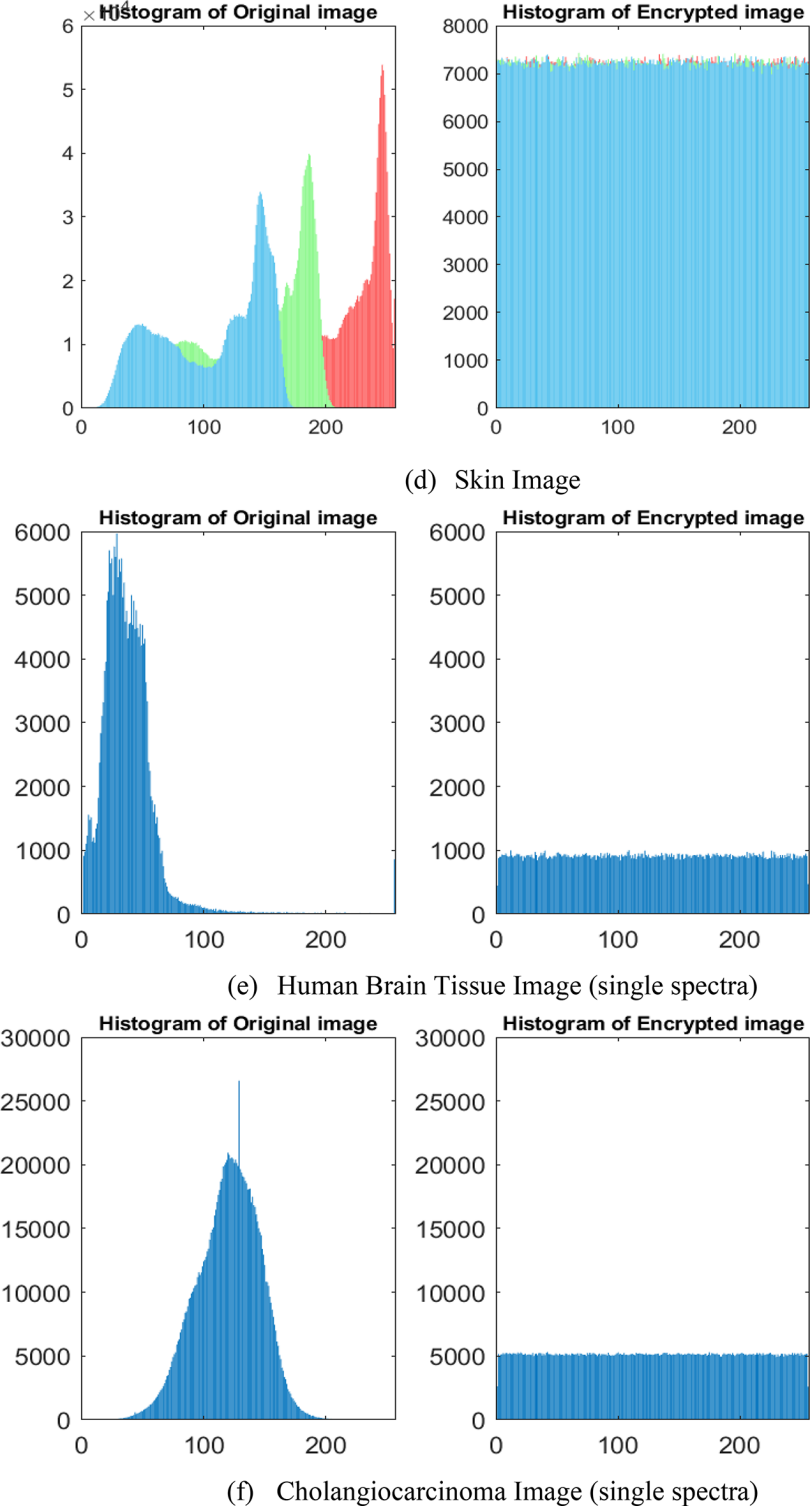
Histogram plots of (**a**,**b**) grayscale, (**c**,**d**) RGB, and (**e**,**f**) Hyperspectral images.

The presence of the relationship between the pixel values of the encrypted image, obtained using the encryption approach is analyzed through statistical methods. This analysis uses techniques such as histogram, variance, chi-square, and correlation coefficient measureo compute the pixel relationships in the encrypted images^39^. Histogram plots are used to visually analyze the uniformity of pixel values in the encrypted images. The uniformly distributed histogram of the encrypted images indicates that attackers cannot infer any information about the original image. Histogram plots of the original, encrypted, and decrypted images are depicted in Fig. 5 to analyze the uniformity of the encrypted images.

From Fig. 5, it can be observed that the pixel values of the encrypted images are uniformly distributed, in contrast to the pixel distribution of the original images. Moreover, the histograms of the original and decrypted images are visually identical, indicating a similarity in the pixel distributions of the decrypted images. The variance and chi-square techniques are used to calculate the uniformity analysis of the encrypted images by analyzing the pixel distribution of the histogram^40^. The mathematical formulation of variance and chi-square is given by Eq. (23) and Eq. (24), respectively.

23 $$\:var\left(x\right)=\frac{1}{{k}^{2}}\:\sum\:_{i=1}^{k}\sum\:_{j=1}^{k}\frac{1}{2}{({x}_{i}-{x}_{j})}^{2}$$

where $\:x$ be the vector of histogram values and $\:x=\left\{{x}_{1},{x}_{2},\dots\:,{x}_{256}\right\}$, $\:{x}_{i}$ and $\:{x}_{j}$ represent the number of pixels with gray values equal to $\:i$ and $\:j$, respectively.

24 $$\:{\chi\:}_{test}^{2}=\sum\:_{i=1}^{M}\frac{({ob}_{i}-e{x}_{i})}{e{x}_{i}}$$

where $\:e{x}_{i}$ is the expected frequency in a uniform distribution which is calculated as $\:e{x}_{i}=\frac{width\times\:length}{256}$. The $\:o{b}_{i}$ is the observed occurrence frequency of each gray level $\:(0-255)$ in the histogram of the encrypted image and $\:M$ represents the number of gray levels.

Table 3 shows the variance and chi-square values of the original as well as encrypted images. The variance of the encrypted images must remain below 5000, and the chi-square value should not exceed 293^40^.

**Table 3:** Analysis of variance and chi-square values for encrypted and original image.

Image name	Variance		Chi-square	
	Original Image	Encrypted Image	Original Image	Encrypted Image
MRI image	4.8732E + 07	1.1431E + 03	8.0152E + 06	188.0190
Ultrasound image	3.5208E + 05	767.2627	8.6830E + 04	189.2200
Diabetic retinopathy image	1.2995E + 07	932.5925	2.5709E + 06	184.5061
Skin image	8.3892E + 07	521.0213	2.9649E + 06	184.1363

**Fig. 6:**
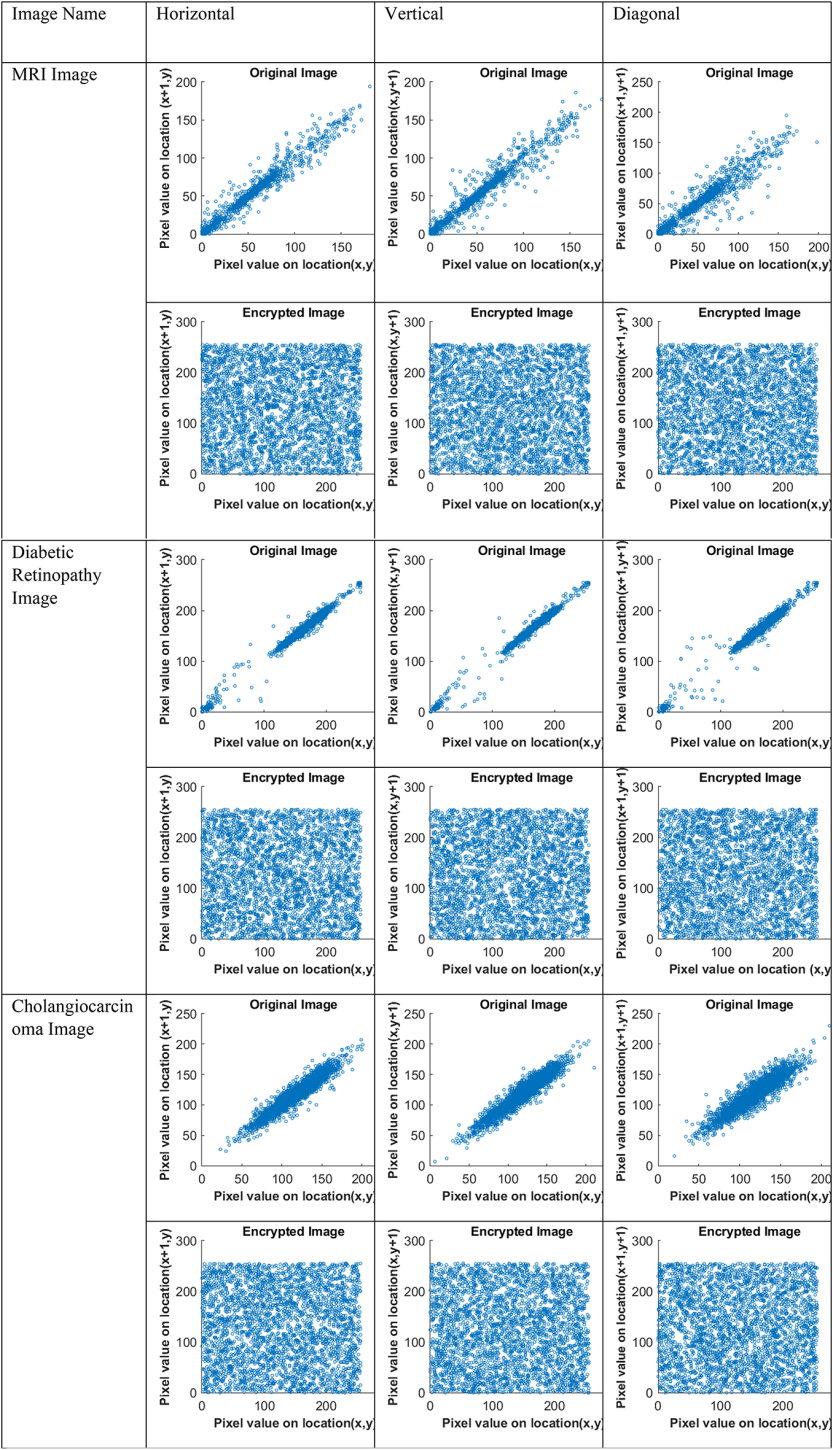
Correlation plots for original and encrypted images.

Table 3 indicates that the encrypted images’ variance and chi-square values meet the required thresholds of 5000 and 293, respectively. The correlation plot analysis is used to visualize the relationship among the encrypted images’ pixel values. In Fig. 6, the correlation plots of the encrypted and original images are shown to depict pixel relationships horizontally, vertically, and diagonally.

The correlation plots of encrypted images are scattered and squared-shaped, showing no negative relationship, which ensures security from information leakage. The correlation coefficients of the original and encrypted images are listed in Table 4.

**Table 4:** Correlation coefficient for original and encrypted images.

Image name	Original image			Encrypted image			
	H	D	V	H	D		V
MRI image	0.9880	0.9853	0.9747	-0.0143		-0.0261	-0.0212
Ultrasound image	0.9762	0.9875	0.9660	0.0294		-0.0009	-0.0035
Diabetic retinopathy image	0.9964	0.9920	0.9706	-0.0074		-0.0166	0.0176
Skin image	0.9875	0.9896	0.9908	-0.0051		0.0176	0.0122
Human brain tissue image	0.9487	0.9622	0.8884	0.0031		0.0336	-0.0337
Cholangiocarcinoma image	0.9533	0.9516	0.9236	-0.0195		-0.0043	-0.0181

The correlation coefficient values of the encrypted images, which are close to zero or negative, indicate that the pixel values in the encrypted images have no relationship with neighboring pixels.

### Differential attack analysis

A number of Pixel Change Rate (NPCR) and Unified Averaged Changed Intensity (UACI) methods are used to analyze the differential attack resistance of the encryption methods^41^. The NPCR and UACI values are calculated by generating two encrypted images, one from the original image and the other by changing a pixel value in the original image. The mathematical formulation of NPCR and UACI is presented by Eqs. (25) and (27) respectively.

25 $$\:N\left({enc}_{1},\:{enc}_{2}\right)=\sum\:_{i,j}\frac{D\left(i,j\right)}{T}\times\:100\:\%$$

where $\:D\left(i,j\right)$ is given by Eq. (26)

26 $$\:D\left(i,j\right)=\left\{\begin{array}{c}0,\:if\:{enc}_{1}\left(i,j\right)={enc}_{2}(i,j)\\\:1,\:if\:{enc}_{1}\left(i,j\right)\ne\:{enc}_{2}(i,j)\:\end{array}\right.$$

$\:{enc}_{1}$ and $\:{enc}_{2}$ are two encrypted images, where $\:{enc}_{1}$ is generated before and $\:{enc}_{2\:}$is generated after a one-pixel change in the original images. $\:T\:$denote the total number of pixels in each encrypted image.

27 $$\:\text{U}\left({\text{e}\text{n}\text{c}}_{1},\:{\text{e}\text{n}\text{c}}_{2}\right)=\sum\:_{\text{i},\text{j}}\frac{\left|{\text{e}\text{n}\text{c}}_{1}(\text{i},\text{j})-{\text{e}\text{n}\text{c}}_{2}(\text{i},\text{j})\right|}{\text{F}\times\:\text{T}}\times\:100\:\text{\%}\:$$

where $\:T$ is the total number of pixels and $\:F$ is the largest supported pixel value of an image of an encrypted image. $\:{enc}_{1}\:$and $\:{enc}_{2}$ are the encrypted images generated before and after the one-pixel change in the original image. For NPCR and UACI to be considered acceptable, their values should exceed 99.60% and 33.40%, respectively^42^. The computed values of the UACI and NPCR for the encrypted images are given in Table 5.

**Table 5:** UACI and NPCR values of encrypted images.

Image name	NPCR (%)	UACI (%)
MRI image	99.6001	33.4620
Ultrasound image	99.6105	33.5191
Diabetic retinopathy image	99.6192	33.4746
Skin image	99.6121	33.4777
Human brain tissue image	99.9970	33.4733
Cholangiocarcinoma image	99.9962	33.4284

The UACI and NPCR values of the encrypted images show that values are within the desired range and successfully resist differential attacks.

### Quantitative analysis

The information entropy, Peak Signal-to-Noise Ratio (PSNR), and Mean Square Error (MSE) metrics are used to quantify the encrypted images generated using the encryption methods^43^. The information entropy is used to measure the degree of unpredictability present in the information content of the encrypted images. For an encrypted image to exhibit pixel randomness, the entropy value should be close to 8 or 16. The MSE and PSNR are used to show discrepancies between the original and encrypted images to measure the image quality. An encryption algorithm having small PSNR values and high MSE values shows the presence of noise in the encrypted image. Table 6 shows the computed results of the metrics for the images.

**Table 6:** Analysis of information entropy, PSNR, and MSE.

Image name	Information entropy		PSNR	MSE
	Original image	Encrypted image		
MRI image	5.4028	7.9997	5.9942	23.7733
Ultrasound image	7.7208	7.9995	8.6597	121.7036
Diabetic retinopathy image	7.3862	7.9996	7.4071	67.8370
Skin image	7.3245	7.9999	8.4595	143.5535
Human brain tissue image	5.3208	15.7855	4.7845	1.4643
Cholangiocarcinoma image	8.3110	15.9643.	6.6008	3.5291E + 03

Table 6 reveals that the proposed encryption approach is extremely efficient due to the small PSNR values and large MSE values. This illustrates the robustness, safety, and efficacy of the propounded encryption approach.

### Key space analysis

The key space analysis is used to check the resistance of the encryption approach against brute-force attacks. The key space of the encrypted approach should be greater than the required $\:{2}^{128}\:$bits^44^. The key space of the propounded approach is $\:{10}^{16\times\:6}={10}^{96}$, as it uses six secret keys, each with a precision of $\:{10}^{16}$. Therefore, the proposed encryption approach is secure from brute force attacks.

### **Comparison with** other state-of-the-art techniques

The performance effectiveness of the ICO-HO algorithm utilized in the proposed encryption method has been evaluated through comparisons with other state-of-the-art optimization algorithms, i.e., PSO^12^, BES^13^, Cheetah Optimization (CO)^45^, Self-adaptive Bald Eagle Search (SABES) optimization^22^, Brown Bear Optimization (BBO)^46^, and Hippopotamus optimization (HO)^24^algorithms. In this evaluation process, only parameter selection of the chaotic maps was made using different optimization algorithms. Table 7 presents the achieved values of the performance metrics (correlation coefficient, entropy, NPCR, UACI) for the DICOM lung CT scan 16-bit images^47^.

**Table 7:** Performance comparison with the other metaheuristic optimization technique.

Methods	Year	Correlation coefficient			Entropy	NPCR	UACI
		H	V	D			
PSO	1995	0.0107	-0.0053	0.0075	15.8063	99.9989	33.3826
BES	2020	-0.0175	0.0090	0.0403	15.8113	99.9989	33.3440
CO	2022	-0.0188	0.0087	0.0315	15.8056	99.9977	33.3291
SABES	2023	-0.0027	-0.0055	-0.0370	15.8113	99.9985	33.3537
BBO	2024	-0.0040	0.0314	-0.0017	15.8118	99.9989	33.3031
HO	2024	0.0196	-0.0288	0.0196	15.8111	99.9989	33.3070
ICO-HO (proposed)	2024	-0.0020	-0.0042	-0.0034	15.8124	99.9969	33.3892

Table 7 shows that the proposed encryption method based on ICO-HO achieved the highest entropy value, which ensures randomness in the pixel values of the encrypted image. Also, all correlation coefficient values, i.e., H, V, and D are lower compared to the other comparative methods, indicating a weak relationship among the pixel values of the encrypted image. The efficacy of the proposed encryption approach is analyzed by comparing the results with existing encryption methods for DICOM lung CT scan images. Entropy, UACI, NPCR, and correlation coefficient metrics are used for the comparison, given in Table 8.

**Table 8:** Comparison with state-of-the-art image encryption techniques.

Author		Year	Correlation coefficient			Entropy	NPCR	UACI
			H	V	D			
Ravichandran et al.^48^ (16 bit)		2021	0.0016	0.0006	0.0022	15.7850	99.6067	33.4700
Meng et al.^49^ (16 bit)		2022	− 0.0080	0.0010	− 0.0074	15.5872	99.9974	33.2819
Muthu and Murli^50^ (16 bit)		2022	− 0.0029	0.0014	0.0012	–	99.9728	33.4782
Wu et al.^51^ (8 bit)		2023	− 0.0164	0.0056	0.0289	7.9993	99.6128	33.5196
Naguib et al.^52^ (8 bit)		2024	0.0013	0.0037	0.0005	7.9878	99.5070	34.4460
Proposed	8 bit	2024	− 0.0032	0.0023	− 0.0014	7.9995	99.6078	33.4517
	16 bit		− 0.0020	− 0.0042	− 0.0034	15.8124	99.9969	33.3892

The proposed encryption method achieves competitive results across key performance metrics. It exhibits low correlation coefficients, ensuring weak pixel correlation among the encrypted pixel values. In the propounded approach, the entropy values are 7.9995 for 8-bit images and 15.8124 for 16-bit images, which are close to their respective ideals. Also, NPCR values were attained using the proposed encryption approach, which showed high sensitivity to pixel changes, while UACI values indicated strong encryption performance. The proposed technique maintains high entropy levels, ensuring that the encrypted images are nearly indistinguishable from random noise, thus providing robust protection against potential attacks and making it comparable to other state-of-the-art techniques.

## Conclusions and future scopes

This paper proposes an adaptive encryption approach based on the optimized chaotic maps. The initial and control parameters of the chaotic maps are optimally selected utilizing the proposed Iterative Cosine Operator-based Hippopotamus Optimization (ICO-HO) algorithm. The efficacy of the ICO-HO algorithm is compared with seven state-of-the-art algorithms namely Hippopotamus Optimization (HO), Whale Optimization Algorithm (WOA), Arithmetic Optimization Algorithm (AOA), Moth Flame Optimization (MFO), Sine Cosine Algorithm (SCA), African Vultures Optimization Algorithm (AVOA), and RIME algorithm (RIME) on CEC-2017 functions. Friedman mean rank analysis gives 1.93, 3.68, 6.17, 7.62, 3.82, 5.89, 4.34, and 2.51 values for ICO-HO, HO, WOA, AOA, MFO, SCA, AVOA, RIME algorithms respectively with p-value 3.56E-24. This shows better performance of the ICO-HO algorithm than other comparative algorithms. The proposed encryption approach is applied to medical images of different modalities and sizes. The effectiveness of the proposed encryption approach is evaluated in RGB, grayscale, and hyperspectral medical images. The effectiveness of the adaptive encryption approach is analyzed using various performance metrics such as visual, histogram, chi-square, variance, NPCR, UACI, correlation coefficient, entropy, PSNR, and MSE. The histogram, chi-square, variance, and correlation coefficient analyses demonstrate the uniform distribution of the pixel values of encrypted images and show the security from the statistical attacks. The UACI and NPCR values for the encrypted images generated using the encryption approach are greater than 33.40% and 99.60%, respectively, which ensure security against differential attacks. The entropy values of 8-bit and 16-bit images are 7.9995 and 15.8124, respectively, which are closer to 8 and 16, which show the randomness of pixel values. Additionally, the propounded encryption approach is compared to the existing encrypted techniques, showing more randomness in the encrypted image than the other encrypted techniques. This work is being extended by designing new hybrid chaotic maps. The proposed ICO-HO algorithm can be applied to other optimization problems like band selection in hyperspectral images.

## Data Availability

All data used for analysis in this research is publically available on the National Institution of Health (NIH) websites (https://openi.nlm.nih.gov/gridquery? it=xg), Cancer imaging archive (https:// wiki.cancerimagingarchive.net/display/Public/CBIS-DDSM), Kaggle (https://www.kaggle. com/), Visual health IT (https://www.visus.com/en/downloads/jivex-dicom-viewer.html), Grand Challenge (https://drive.grand-challenge.org/DRIVE/), and University Medical Center Groningen (UMCG) website (http://www.cs.rug.nl/~imagi ng/databases/melanoma_naevi/).
